# Is thyroid excision mandatory with laryngectomy in carcinoma larynx?

**DOI:** 10.1186/s12885-020-07205-5

**Published:** 2020-07-28

**Authors:** Surendra Singh Baghel, Pawan Singhal, Namita Verma, Ritu Sehra, Rajeev Yadav, Sunita Agarwal, Man Prakash Sharma, D. P. Gupta

**Affiliations:** 1 Department of Neuro-otology, Sanjay Gandhi Post Graduate Institute, Lucknow, Uttar Pradesh India; 2 Department of ENT, SMS Medical College, Jaipur, 302004 Rajasthan India; 3 Department of Preventive and Social Medicine, Department of Otorhinolaryngology, SMS Medical College, Jaipur, Rajasthan India

**Keywords:** Laryngeal carcinoma, Thyroid invasion, Malignancy larynx, Thyroidectomy

## Abstract

**Background:**

Advanced stage operable cancers of larynx are treated with total laryngectomy including thyroid resection in most of the cases, which may expose patient to hypothyroidism and hypoparathyroidism. The requirement of thyroidectomy during Total Laryngectomy is controversial.

**Methods:**

A cross sectional observational study was set out to review preoperative clinical and radiological assessment; intraoperative and histopathological findings; and follow-up data to predict thyroid gland invasion in the setting of squamous cell carcinoma of the Larynx.

**Results:**

11 (16%) out of 69 patients had thyroid gland involvement on histopathological examination with mean age 63 years. Out of these 11 cases, 8 (72%) underwent primary total laryngectomy. 90% patients with thyroid gland involvement were male. 9 cases with thyroid gland involvement were staged as T4a preoperatively.

**Conclusion:**

Invasion of thyroid gland by laryngeal cancer is uncommon. Unnecessary hemithyroidectomies lead to hypothyroidism and hypoparathyroidism. The study points out the clear indications of thyroid excision in patients undergoing total laryngectomy. We can suggest that total thyroidectomy should be done with total laryngectomy in cases which have gross clinical, radiological or intraoperative thyroid gland involvement, subglottic extension and thyroid cartilage invasion. This can save the patients from the brunt of unnecessary morbid hypothyroidism and hypoparathyroidism.

## Background

Ipsilateral hemithyroidectomy or total thyroidectomy are considered mandatory for all patients undergoing total laryngectomy (TL) for squamous cell carcinoma (SCC) of the larynx. This is because the anatomic position of the thyroid gland renders it vulnerable to involvement in advanced laryngeal cancers. Thyroid gland being an adjacent organ to larynx, can be contiguously invaded due to the presence of anatomical zones of susceptibility (cricothyroid membrane) and resistance (laryngeal cartilages) or a non-contiguous spread can occur lympho-vascularly. This explains why laryngeal carcinomas may be associated with thyroid gland metastasis [1–4].

It is well documented that patients are subjected to hypothyroidism as a consequence of head and neck cancer operations, especially for laryngeal carcinoma. Additionally, patients are at risk for complications of thyroidectomy as well as risks for laryngectomy and neck dissection, when thyroid gland is included in surgery for laryngeal carcinoma. Therefore, the decision of thyroidectomy is important for the management of laryngeal carcinoma [5–7]. This study examines preoperative clinical, radiological and histopathologic characteristics that can be used to predict thyroid gland invasion in the setting of SCC of the Larynx and can guide whether thyroid gland excision is necessary or not in all total laryngectomies.

## Methods

This hospital based cross sectional observational study was carried out in the Department of Otorhinolaryngology & Head & Neck Surgery at SMS Medical College & Hospital, Jaipur during June 2015 to December 2016 after due permission from Ethics Committee and Research and Review Board of the institution. The sample size required was 69 laryngeal cancer cases at 95% of confidence and 10% absolute allowable error to verify the expected 22% proportion of thyroid gland involvement in cases of carcinoma larynx. All the cases were staged according to American Joint Committee on Cancer, 8th edition. Tumour extent, size, subsite involved, thyroid involvement and differentiation were based on the gross and histological analysis of pathological specimen. All biopsy proven cases of squamous cell carcinoma of larynx were subjected to total laryngectomy with hemi or total thyroidectomy, with or without partial pharyngectomy and the tissue samples were sent for histopathological examination. We reviewed the histopathological reports of all the patients.

The Patients included in the study, were cases of laryngeal carcinoma of stage T3 and stage T4; candidate for total laryngectomy with or without preoperative chemoradiotherapy. Those patients were excluded who had laryngeal carcinoma stage TI, T2 and T4b, with previous thyroidectomy, history of primary thyroid cancer, candidate for conservative laryngectomy, with distant metastasis and comorbidities precluding major surgical intervention.

Prospective data including radiological findings on Contrast enhanced computerised tomography scans (CECT) of neck of all patients fulfilling the above-mentioned criteria was collected and correlated with histopathological findings to find any association between the two.

## Results

The age range of patients studied was 40–70 years, with mean age of 54 years. Out of 69 patients, 63 were males and 6 females. There were more patients in age group 55–70 years (58%). Majority of the patients presented with transglottic (38) involvement, followed by glottis (16), supraglottis (10) and subglottis (5) lastly. According to clinical, radiological and pathological examination, 20 were of T3 while T4a was seen in 49 cases (Table 1).

**Table 1:** Clinico-epidemiological data of our study

S. No.	Variables	Total Number(N)	Thyroid Gland Invasion(N/%)	No Thyroid Gland Invasion(N/%)	‘***p***’ value*
1	**Gender**				
	Male	63	10 (90.9)	53 (91.4)	0.594
	Female	6	1 (9.1)	5 (8.6)	
2	**Site of Tumour**				
	Transglottic	38	8 (72.7)	30 (51.7)	0.153
	Glottic	16	1 (9)	15 (25.9)	
	Supraglottic	10	0 (0)	10 (17.2)	
	Subglottic	5	2 (18.2)	3 (5.2)	
3	**T Staging**				
	T3	20	2 (18.2)	18 (31)	0.618
	T4a	49	9 (81.9)	40 (68.9)	
4	**Surgery (Total Laryngectomy)**				
	With Hemithyroidectomy	50	0 (0)	50 (86.2)	< 0.001
	With Total Thyroidectomy	19	11 (100)	8 (13.8)	
5	**Type of Laryngectomy**				
	Total Primary Laryngectomy	54	8 (72.7)	46 (79.3)	0.931
	Salvage Total Laryngectomy	15	3 (27.3)	12 (20.7)	

*Chi-square test

Present study found that only 11 out of 69 patients undergoing hemi/total thyroidectomy had thyroid gland invasion, i.e. Fifty-eight patients (84%) didn’t have thyroid gland invasion. According to the results, the risk of metastasis in thyroid gland increases 5.5 times when subglottic extension is found clinically and increases up to 8.8 times when thyroid cartilage invasion is also present clinically. Similarly, on radiological and intra operative evaluation, all the patients found to have gross thyroid gland involvement were later confirmed to have disease in histopathology.

One more interesting finding was that 6 patients of total 69 patients had metastasis in thyroid gland even in the absence of gross thyroid gland involvement either on clinical, radiological or intra operative examination. This number reduced to 2 and 4 in the absence of subglottic extension and thyroid cartilage involvement either on clinical, radiological or intra operative evaluation. Therefore, it is an eye-opening finding that only very few numbers of cases were having confirmed disease on histopathology in thyroid gland in the absence of gross thyroid gland involvement, thyroid cartilage involvement and subglottic extension on clinical, radiological and intra operative evaluation.

The negative predictive value of clinical examination, radiological evaluation and intra operative assessment were 93, 84 and 95% respectively. It can be inferred from above results that if we rely on clinical examination, radiological assessment and intra operative evaluation, almost all patients without involvement of thyroid gland can be salvaged from unnecessary trauma to thyroid gland. Overall, the negative predictive value was 87% that means in the absence of gross thyroid gland involvement at any level of examination, we can save unnecessary trauma to thyroid gland in most of the patients. Negative predictive value for subglottic extension and thyroid cartilage involvement was 91 and 80% respectively which was again a good indicator to avoid unnecessary thyroidectomies among patients without metastasis in thyroid gland.

The present study recommends highest accuracy of metastatic disease in thyroid gland if gross thyroid gland involvement is there on clinical, radiological and intra operative evaluation (100%) and highest surety of non-involvement of thyroid gland if there is no subglottic extension found on clinical, radiological and intraoperative evaluation.

## Discussion

Laryngeal carcinoma is one of the most common head and neck cancers found in both men and women. Most carcinomas arise in the glottic region and spread via direct extension to invade adjacent laryngeal structures. Extra laryngeal extension into structures such as the thyroid gland occurs late. The incidence of invasion of the thyroid gland by laryngeal carcinomas is reported to be between 0 and 30% [1, 8–11].

The thyroid gland may be involved by direct invasion of the tumour because of its close proximity to the structures, as in post-cricoid lesions, direct invasion in transglottic or supraglottic carcinomas because of infiltration of the tumour through the thyroid cartilage or through the cricothyroid membrane, secondary involvement in subglottic/transglottic cancers via lymphatic spread to the pre-laryngeal lymph node or to the lower deep cervical lymph nodes and large anterior commissure lesions also cause invasion of the thyroid gland [12–14].

In 1955, Ogura recommended routine ipsilateral hemithyroidectomy and isthmectomy for all total laryngectomy cases to ensure adequate local control of tumour [15]. In 1973, Harrison reiterated that total laryngectomy should always include at least isthmectomy and ipsilateral lobectomy plus frozen sectioning of the contralateral lobe [13].

The incidence of hypothyroidism and hypoparathyroidism after total laryngectomy with hemi or total thyroidectomy is relatively common and increases with time and postoperative radiotherapy [6, 16–20].

The incidence of hypothyroidism varies with different methods used for treatment of carcinoma larynx. Total laryngectomy alone has an incidence of hypothyroidism of 20 to 63% [2, 16]. Total laryngectomy with hemithyroidectomy but no radiotherapy is associated with incidence of hypothyroidism in 23% cases. Solely radiotherapy rendering the thyroid gland at risk of hypothyroidism in 40–50% of cases [16, 21]. Although the incidence of hypothyroidism in patients treated with a total laryngectomy and adjuvant radiotherapy without thyroidectomy is reported as low as 38%, but when hemithyroidectomy is done along, the incidence increases up to 59–89% [2, 7, 16, 20] (Table 2).

**Table 2:** Incidence of hypothyroidism in various modalities used for carcinoma larynx treatment

S. No.	Therapeutic modalities	Incidence of hypothyroidism (%)
1.	Total laryngectomy alone	20–63
2.	Total laryngectomy with hemithyroidectomy (no radiotherapy)	23
3.	Total laryngectomy with adjuvant radiotherapy (no thyroidectomy)	38
4.	Total laryngectomy with hemithyroidectomy and adjuvant radiotherapy	59–89
5.	Radiotherapy alone	50

(Note: Data collected from various studies)

Hypothyroidism is a common complication in treated head and neck carcinoma patients which may remain undiagnosed and uneventful if thyroid function testing is not performed. Patients seldom recognize early symptoms such as weight gain, cold intolerance, dry skin and constipation. Usually the only complaint is fatigue and patients consider it as being a normal adverse event of the treatment. Due to the slow development and relatively non-specific nature of the symptomatology, a considerable number of patients may possibly have undiagnosed hypothyroidism [19].

In recent years, there has been an evolving trend toward the use of “organ-sparing” modalities for the treatment of selected patients with head and neck cancers [22] This strategy seeks to preserve anatomical structures in the hope that they will continue to provide form and function to laryngeal cancer patients after treatment [23].

In our study, majority of the patients belonged to age group 55–70 years with the mean age at presentation was found to be 54.6 years. Hegazy et al. [24] conducted a study in 40 patients of age 45 to 80 years with mean age of 61.1 years. Nayak et al. [25] conducted a study in 45 patients with mean age of 54.04 years. Hence our study concurs with other studies as far as age is considered.

In the present study, the number of male patients were 63 and female patients were 6. Hegazy et al. [24] observed 37 males (92.5%) and 3 females (7.5%) in his study. In the study conducted by Nayak et al. [25], the number of male patients were 44 while number of female patients was 1. There was male predominance in our study which is in agreement with other studies.

We observed that patients with supraglottic involvement were 10 (14.4%) while glottic involvement was seen in 16(23.1%), sub glottic involvement was seen in 5 patient (7.2%) and patient with transglottic involvement was found to be 38(55.01%). Nayak et al. [25] found the number of patients with supra glottic involvement was 2(4.4%), glottic involvement was 9 (20%), subglottic 1 (2.2%) while transglottic involvement was seen in 33 patients (73.3%). Thus, the majority of patients observed were found to have transglottic involvement.

The presented study had 29 (42.02%) patients with preoperative staging T3 while the number of patients with T4a staging was 40 (57.98%). In the study conducted by Hegazy et al. [24] number of patients with T3 stage were 18(45%) and patients with T4a stage were 22(55%). Hence majority of the cases were in T4a stage preoperatively as in our study.

In our study of 69 cases, 54 patients were operated as primary total laryngectomy (78.27%) while patients who were operated as salvage laryngectomy were 15(21.73%). In the study done by Nayak et al. [25] patients operated as primary total laryngectomy were 41 (91%) while patients operated as salvage laryngectomy were 4 (9%). In his study of 52 patients, Ohad Hilly [26] operated 14 (27%) patients as primary total laryngectomy and 38 (73%) as salvage laryngectomy. Thus, most of the patients of our study operated as primary total laryngectomy as appears in other studies.

The number of patients operated for hemithyroidectomy with total laryngectomy were 50 (72.46%) while total thyroidectomy with total laryngectomy were 19 (27.54%) in this study. In the study of Hegazy et al. [24] number of patients who were operated as hemi thyroidectomy with total laryngectomy were 33 (82.5%), those operated as subtotal thyroidectomy with total laryngectomy 4(10%) and who were operated as total thyroidectomy with total laryngectomy were 3(7.5%). Ohad hilly [26] found the number of patients who were operated as hemi thyroidectomy with total laryngectomy were 45 (86.54%) while who were operated as total thyroidectomy with total laryngectomy were 7 (13.46%). Thus, the majority of patients were operated as hemi thyroidectomy with total laryngectomy.

In the present study, 11(15.94%) cases were found to have thyroid gland involvement histopathologically, 3(4.3%) cases clinically while on radiological examination we rightly predicted 5(7.24%) cases who showed thyroid gland involvement. In study conducted by Hegazy et al. [24] out of 40 patients, thyroid gland involvement was found in 4(10%) cases histopathologically and in 2(5%) cases radiologically. Similarly, Ohad Hilly [26] found thyroid gland involvement in 11(21%) cases out of 52. Out of these 11, thyroid gland was radiologically involved in only 1 case (9.09%). Nayak et al. [25] observed thyroid gland involvement in 5 cases (11.11%) out of 45 patients.

In this study, out of those 11 cases with thyroid involvement, 8 cases had transglottic extension (72.72%) while 2 cases had subglottic involvement (18.18%) and 1 patient had only glottic involvement (9.09%). In the study by Hegazy et al. [24], 3 out of 4 (75%) cases had transglottic involvement while 1 case (25%) had only glottis involvement. In study by Nayak et al. [25], all the cases presented with transglottic involvement. Out of 11 cases with thyroid involvement 9 cases (81.81%) were in preoperative T4a stage while 2 (18.18%) cases were in T3 stage. Hegazy et al. [24] reported all the cases as T4a.

In the present study, out of the 54 patients who were operated as primary total laryngectomy, 8 cases showed thyroid involvement while in cases of salvage laryngectomy thyroid involvement was seen in 3 cases. Hemithyroidectomy was performed in 50 cases which showed no thyroid involvement. Total thyroidectomy was performed in 19 cases out of which thyroid involvement was seen only in 11 cases. In study by Hegazy et al. [24] hemithyroidectomy was performed in 33 cases (82.5%) which showed no thyroid involvement, subtotal thyroidectomy was performed in 4 cases in which 1 case showed thyroid involvement and in 3 cases (7.5%) of total thyroidectomy thyroid involvement was seen in all 3 cases.

In the present study, all the patients with thyroid gland involvement had fixed vocal cord and anterior commissure involvement whereas 81.81% of the patients had subglottic extension and arytenoids fixation. Hegazy et al. [24] observed that all the cases with thyroid gland involvement had unilateral fixed vocal cord. All the cases had infiltrated anterior commissure and unilateral subglottic extension > 1 cm (Table 3).

**Table 3:** Comparison of various studies on Thyroid Gland Involvement in Advanced Laryngeal Squamous Cell Carcinoma

S. No.	Author	N	Incidence of TGI
1.	Dadas B et al. (2001) [27]	182	1%
2.	Asher A. Mendelson et al. (2009) [23]	399	8%
3.	Ohad Hilly et al. (2011) [26]	52	21%
4.	L. Gaillardina et al. (2012) [28]	87	12.6%
5.	Nayak et al. (2012) [25]	45	11.1%
6.	Kumar R. et al. (2013) [29]	1287	10.7%
7.	Moustafa Mourad et al. (2015) [30]	343	2%
8.	Hegazy et al. (2015) [24]	40	10%
9.	Mangussi-Gomes et al. (2016) [31]	83	18.1%
10.	Jan Warren A. Holgado et al. (2017) [32]	118	11%
11.	Maria Concepcion F. Vitamog et al. (2017) [33]	61	1.6%
12.	Our study	69	15.9%

(*N* Total number of cases, *TGI* Thyroid gland invasion)

On radiological assessment, only frank thyroid gland invasion could predict the thyroid gland involvement but only in 5 cases (7.24%) in our study. Hegazy et al. [24] could diagnose thyroid gland involvement in 2 cases (50%) only on the basis of preoperative CECT finding of gross thyroid gland invasion.

Intraoperatively thyroid cartilage involvement, growth abutting thyroid gland and gross invasion of thyroid gland was found in 63.63, 45.45, 45.45% respectively. All these were statistically significant (*p* < 0.001) making them important markers for predicting thyroid gland involvement intraoperatively.

As the present study is a single centre study which mostly takes care of a particular social class, there is a limitation of selection bias which is inevitable and may enter in the study.

## Conclusion

Invasion of thyroid gland by laryngeal cancer is uncommon. However, ipsilateral hemithyroidectomy is routinely performed during total laryngectomy (TL) for laryngeal cancers while hemithyroidectomies are also associated with hypothyroidism in 23–63% and hypoparathyroidism in 25–52% cases [25]. Based on our observation and its statistical significance we can suggest that thyroidectomy should be done with total laryngectomy in cases which have clinical, radiological or gross intraoperative thyroid gland involvement, subglottic extension and thyroid cartilage invasion. This can save the patients from the brunt of unnecessary morbid hypothyroidism and hypoparathyroidism. It is also suggested that multicentric studies on large sample sizes should be encouraged to find out some score/index to predict involvement of thyroid gland on the basis of clinical, radiological and intra operative evaluation to avoid undue removal of total/partial thyroid gland and offer good quality of life to those patients in which disease is not extended up to thyroid gland.

## Data Availability

We promise to that the materials described in the manuscript, including all relevant raw data, will be freely available to any scientist wishing to use them for non-commercial purposes, without breaching participant confidentiality. The datasets used and/or analysed during the current study are available from the corresponding author on reasonable request.

## Abbreviations

TL: Total laryngectomy
SCC: Squamous cell carcinoma
CECT: Contrast enhanced computerised tomography scan
TGI: Thyroid gland invasion

## References

[1] Sparano A, Chernock R, Laccourreye O (2005). Predictors of thyroid gland invasion in glottic squamous cell carcinoma. Laryngoscope.

[2] Biel MA, Maisel RH (1985). Indications for performing hemithyroidectomy for tumours requiring total laryngectomy. Arch Otolaryngol Head Neck Surg.

[3] Brennan JA, Meyers AD, Jafek BW (1991). The intraoperative management of the thyroid gland during laryngectomy. Laryngoscope.

[4] Elliot MS, Odell EW, Tysome JR (2010). Role of thyroidectomy in advanced laryngeal and pharyngolaryngeal carcinoma. Otolaryngol Head Neck Surg.

[5] Smolarz K, Malke G, Voth E, Scheidhauer K, Eckel HE, Jungehulsing M (2000). Hypothyroidism after therapy for larynx and pharynx carcinoma. Thyroid.

[6] Lo Galbo AM, de Bree R, Kuik DJ, Lips PT, Mary B, Von Blomberg E, Langendijk JA, Leemans CR (2007). The prevalence of hypothyroidism after treatment for laryngeal and hypopharyngeal carcinomas: are autoantibodies of influence?. Acta Otolaryngol.

[7] Sinard RJ, Tobin EJ, Mazzaferri EL, Hodgson SE, Young DC, Kunz AL, Malhotra PS, Fritz MA, Schuller DE (2000). Hypothyroidism after treatment for nonthyroid head and neck cancer. Arch Otolaryngol Head Neck Surg.

[8] Ceylan A, Koybasioglu A, Yilmaz M (2004). Thyroid gland invasion in advanced laryngeal and hypopharyngeal carcinoma. Kulak Burun Bogaz Ihtis Derg.

[9] Gallegos-Hernández JF, Minauro-Munoz G, Hernández-Hernández DM (2005). Thyroidectomy associated with laryngectomy in laryngeal carcinoma treatment. Is it routinely necessary?. Cir Cir.

[10] Croce A, Moretti A, Bianchedi M (1991). Thyroid gland involvement in carcinoma of the larynx. Acta Otorhinolaryngol Ital.

[11] Gilbert RW, Cullen RJ, van Nostrand AW (1986). Prognostic significance of thyroid gland involvement in laryngeal carcinoma. Arch Otolaryngol Head Neck Surg.

[12] Harrison DFN (1970). Pathology of pharyngeal cancer in relation to surgical management. J Laryngol Otol.

[13] Harrison DFN (1973). Thyroid gland in the management of laryngopharyngeal cancer. Arch Otolaryngol.

[14] Kirchner JA (1977). Two hundred laryngeal cancers patterns of growth and spread as seen in the serial section. Laryngoscope.

[15] Ogura JH (1955). Surgical pathology of cancer of the larynx. Laryngoscope.

[16] Thorp MA, Levitt NS, Mortimore S (1999). Parathyroid and thyroid function five years after treatment of laryngeal and hypopharyngeal carcinoma. Clin Otolaryngol Allied Sci.

[17] Ho AC, Ho WK, Lam PK (2008). Thyroid dysfunction in laryngectomees—10 years after treatment. Head Neck.

[18] Garcia-Serra A, Amdur RJ, Morris CG (2005). Thyroid function should be monitored following radiotherapy to the low neck. Am J Clin Oncol.

[19] Léon X, Gras JR, Pérez A (2002). Hypothyroidism in patients treated with total laryngectomy. A multivariate study. Eur Arch Otorhinolaryngol.

[20] Cinar U, Yigit O, Alkan S (2003). The effect of laryngectomy and postoperative radiotherapy on thyroid gland functions. Kulak Burun Bogaz Ihtis Derg.

[21] Aich RK, Ranjan DA, Pal S (2005). Iatrogenic hypothyroidism: a consequence of external beam radiotherapy to the head & neck malignancies. J Carcinoma Res Ther.

[22] Pfister DG, Laurie SA, Weinstein GS (2008). American Society of Clinical Oncology clinical practice guideline for use of larynx–preservation strategies in the treatment of laryngeal cancer. J Clin Oncol.

[23] Mendelson AA, Al-Khatib TA, Julien M, Payne RJ, Black MJ, Hier MP (2009). Thyroid gland management in total laryngectomy: meta-analysis and surgical recommendations. Otolaryngol Head Neck Surg.

[24] Mohamed AH, Mohamed SH, Ahmed FA, Osama MF. Thyroid gland invasion in T3 and T4 laryngeal carcinoma. Med J Cairo Univ. 2015;83(2):117–22.

[25] Nayak SP, Singh V, Dam A, Bhowmik A, Jadhav TS, Ashraf M, Shah RK, Biswas J (2013). Mechanism of thyroid gland invasion in laryngeal cancer and indications for thyroidectomy. Indian J Otolaryngol Head Neck Surg.

[26] Hilly O, Raz R, Vaisbuch Y, Strenov Y, Segal K, Koren R, Shvero J (2012). Thyroid gland involvement in advanced laryngeal cancer: association with clinical and pathologic characteristics. Head & neck.

[27] Dadas B, Uslu B, Cakir B, Ozdogan HC, Calis AB, Turgut S (2001). Intraoperative management of the thyroid gland in laryngeal cancer surgery. J Otolaryngol.

[28] Gaillardin L, Beutter P, Cottier JP, Arbion F, Moriniere S (2012). Thyroid gland invasion in laryngopharyngeal squamous cell carcinoma: prevalence, endoscopic, and CT predictors. Eur Ann Otorhinolaryngol Head Neck Dis.

[29] Kumar R, Drinnan M, Robinson M (2013). Thyroid gland invasion in total laryngectomy and total laryngopharyngectomy: a systematic review and meta-analysis of the English literature. Clin Otolaryngol.

[30] Mourad M, Saman M, Sawhney R, Ducic Y (2015). Management of the thyroid gland during total laryngectomy in patients with laryngeal squamous cell carcinoma. Laryngoscope.

[31] Mangussi-Gomes J, Danelon-Leonhardt F, Moussalem GF, Ahumada NG, Oliveira CL, Hojaij FC. Thyroid gland invasion in advanced squamous cell carcinoma of the larynx and hypopharynx. Braz J Otorhinolaryngol. 2017;83(3):269–75. 10.1016/j.bjorl.2016.03.019 PMC9444747 27209377

[32] Holgado JW, Grullo PE, Gloria JD, Pontejos AQ (2017). Thyroid gland involvement in advanced laryngeal squamous cell carcinoma. Acta Med Philipp.

[33] Vitamog MC, Castañeda SS (2017). Thyroid gland invasion in laryngeal carcinoma. Philipp J Otolaryngol-Head Neck Surg.

